# An Innovative Probiotic-Based Supplement to Mitigate Molecular Factors Connected to Depression and Anxiety: An In Vitro Study

**DOI:** 10.3390/ijms25094774

**Published:** 2024-04-27

**Authors:** Sara Ferrari, Simone Mulè, Giorgia Rosso, Francesca Parini, Rebecca Galla, Claudio Molinari, Francesca Uberti

**Affiliations:** 1 Laboratory of Physiology, Department for Sustainable Development and Ecological Transition, 13100 Vercelli, Italy20032921@studenti.uniupo.it (F.P.); claudio.molinari@med.uniupo.it (C.M.); 2 Noivita Srls, Spin Off of the University of Piemonte Orientale, Via Solaroli 17, 28100 Novara, Italy

**Keywords:** oral probiotic formulation, combined effect, in vitro study, mental disorder, gut–brain axis

## Abstract

The gut–brain axis is a bidirectional relationship between the microbiota and the brain; genes related to the brain and gut synaptic formation are similar. Research on the causal effects of gut microbiota on human behavior, brain development, and function, as well as the underlying molecular processes, has emerged in recent decades. Probiotics have been shown in several trials to help reduce anxiety and depressive symptoms. Because of this, probiotic combinations have been tested in in vitro models to see whether they might modulate the gut and alleviate depression and anxiety. Therefore, we sought to determine whether a novel formulation might affect the pathways controlling anxiety and depression states and alter gut barrier activities in a 3D model without having harmful side effects. Our findings indicate that *B. bifidum* novaBBF7 10 mg/mL, *B. longum* novaBLG2 5 mg/mL, and *L. paracasei* TJB8 10 mg/mL may influence the intestinal barrier and enhance the synthesis of short-chain fatty acids. Additionally, the probiotics studied did not cause neuronal damage and, in combination, exert a protective effect against the condition of anxiety and depression triggered by L-Glutamate. All these findings show that probiotics can affect gut function to alter the pathways underlying anxiety and depression.

## 1. Introduction

The gut–brain axis, a two-way relationship between the microbiota and the brain, has come to be recognized, as the two have significant effects on one another [1]. This is possibly because brain and intestinal tract genes are similar, especially in relation to neuronal synapse formation. Thus, some gene mutations can lead to brain and gut abnormalities [2]. It is also known that the brain and the microbiota communicate through several systems, such as the immune system, tryptophan metabolism, the vagus nerve, and the enteric nervous system, which uses microbial metabolites like short-chain fatty acids, branched-chain amino acids, and peptidoglycans that function as neurotransmitters. This is important for numerous physiological processes, including cognitive ones. Thus, the significance of the gut–brain axis in the fields of researching the molecular and physiological foundation of mental and neurodegenerative illnesses is becoming increasingly clear [3].

Furthermore, in recent decades, research has reported the causal impacts of gut microbiota (GMB) on human behavior, development, and function of the brain, as well as understanding the underlying molecular mechanisms [4,5]. Indeed, GMB has been linked to the growth and operation of immune cells in the central nervous system (CNS), including microglia. For example, it has been observed that germ-free mice have less developed microglia than controls, which increases the risk of exposure to pathogens. Moreover, it has been demonstrated that antibiotic treatment caused microglia to regress into an immature state, indicating that ongoing GMB signaling is necessary for microglial activity [6]. Thus, it is simple to understand how human health may be severely impacted if the dynamic relationship between the host and its GMB is disrupted [7].

Moreover, GMB is required for the immune system to operate properly, and immunological dysfunction is related to Schizophrenia (SCZ). As a result, changes in the microbiota could have an important role in the development and therapeutic response of SCZ caused by bacterial infections, like lower levels of the taxa *Roseburia* and *Faecalibacterium*, contributing to a chronic inflammatory state [8,9]. Notably, both species have a significant role in ensuring the integrity of the intestinal barrier by producing butyrate [10]. Butyrate, serotonin (5HT), norepinephrine, dopamine, and aminobutyric acid are only a few of the specific chemicals the GMB can produce on its own or in conjunction with other organs [11].

Analysis of the microbiota–gut–brain axis and the effects that bacteria in the microbiota may have on the functioning of the CNS has shown that, for psychiatric disorders, an exciting approach is probiotic supplementation [12]. Probiotics, which are live microorganisms that provide a health advantage to the host when given in sufficient quantities, have the ability to alleviate mood disorders by transmitting signals to the brain through the vagal nerve [13]. Indeed, in humans, probiotics can lower proinflammatory cytokines and oxidative stress and alleviate symptoms of depression and anxiety [13]. In this context, it was discovered that rats given probiotics had better results than those given a placebo. This result was similar to that when rats are given diazepam, the standard reference substance when performing a test on the degree of anxiety [14]. Based on scientific evidence, probiotics, including *Bifidobacteria* and/or *Lactobacillus* spp., have been shown to alleviate depression by relieving its symptoms [15,16]. Along with this, depression-related symptoms are also regarded as prevalent clinical signs in SCZ patients and have been linked to poor clinical outcomes [17].

Furthermore, a probiotic combination of *Lactobacillus rhamnosus* and *Bifidobacterium animalis* was able to reduce the acute phase reactant von Willebrand factor in SCZ patients, which is thought to be a side effect of probiotic-induced enhancement of intestinal epithelial integrity [18,19].

Moreover, in the SCZ condition, some examinations demonstrated that utilizing probiotics, antibiotics, and antimicrobials caused no significant improvement in negative symptoms of SCZ. Still, when combined with vitamin D in one trial, there was a trend toward efficacy vs. placebo [20]. Neuropsychiatric conditions such as depression and schizophrenia may also be affected by variations in microbial substrates and exogenous probiotics [21]. It was found that probiotic treatment for 14 weeks reduced the common somatic symptoms of schizophrenia but had no discernible effect on the positive and negative symptom scale (PANSS) [18].

Certain nutritional therapies, such as probiotics and vitamin D, have the potential to augment minor symptoms of neuropsychiatric disorders or yield ineffective outcomes [18,22].

Considering this evidence, probiotics are progressively gaining prominence in psychiatry, and their role as psychobiotics has become increasingly remarkable [1]. The term psychobiotics was coined a few years ago to describe a novel class of probiotics that are capable of producing chemicals that can impact the gut–brain connection, enhance mood, alleviate anxiety and depression, and provide numerous other advantages [23]. Indeed, patients with mental illness who follow drug therapies have a greater probability of metabolic syndrome than the general population. Furthermore, the use of antipsychotics, such as olanzapine and clozapine, increases the occurrence of metabolic side effects, type 2 diabetes, and antipsychotic-induced weight gain (AIWG) [24]. Moreover, it was found that olanzapine-induced weight increase in germ-free mice was required and was sufficient for developing a GMB. Thus, it was postulated that olanzapine affects AIWG via in vitro antibacterial action against resident intestinal bacteria [25]. In addition, olanzapine can modify the GMB profile in both male and female rats, especially increasing *Firmicutes* and decreasing *Bacteriodetes* [26]. These findings have allowed for an evolution in focus toward novel therapeutics that concentrate on the favorable relationship between probiotics and anxiety, depression, and mood disorders [23]. In this context, *Lactobacillus plantarum* is a significant testimonial; an injection of *L. plantarum* PS128 (PS128) reduced anxiety and depression-like behaviors in mice.

Furthermore, *L. plantarum* decreased inflammation and corticosterone levels considerably. Compared to control mice, *L. plantarum* PS128 treatment significantly enhanced dopamine and serotonin levels in the prefrontal cortex and striatum [27]. Also, the daily administration of *L. plantarum* may lead to a decrease in depressive symptoms and an improvement in deep sleep quality [28]. Discovering the first proof of the positive impact of probiotics *B. longum* R0175 and *L. helveticus* R0052 on rats’ post-myocardial infarction depression was critical. This beneficial probiotic effect contributed to preserving the integrity of the intestinal barrier and may have been related to the host’s inflammatory condition after the infarct [29]. Further evidence that probiotics may be a viable treatment option for mood disorders showed that two strains of *Bifidobacterium* improved the anxiety phenotype of intrinsically anxious BALB/c mice in a manner specific to that strain, and that this effect outperformed that of the antidepressant escitalopram [30]. Furthermore, a substantial reduction in “Beck Depression Inventory” scores was observed among depressed patients who were administered a probiotic combination of *Bifidobacterium longum* and *Lactobacillus helveticus*, as opposed to those who received a placebo or prebiotic treatments [31]. Furthermore, in addition to *Bifidobacterium bifidum*, *Bifidobacterium lactis*, and *Lactobacillus acidophilus*, *Bifidobacterium longum* has recently been applied to therapeutic purposes in a randomized experiment in conjunction with the anti-depressive drug sertraline, and according to the results, the efficacy of sertraline-probiotic supplementation was more significant than a control on anxiety symptoms [32].

Considering that there are findings in older adults in which depressive and anxiety symptoms are associated with an increased risk of cognitive impairment and neurodegenerative disorders [33], we sought to verify the effects of several probiotics tested in cognitive decline to ameliorate mood disorders [34]. Therefore, this investigation aimed to test three probiotic strains as previously described, first using a gut in vitro model that resembles oral consumption and then examining the strains’ effects on the underlying processes of a model that mimics mood disorders to validate this assumption. In particular, we decided to test whether the combination explored to ameliorate cognitive decline may reduce symptoms in mood disorders, including anxiety and depression. For that, the study explores the ability of *B. bifidum* novaBBF7, *B. longum* novaBLG2, and *L. paracasei* TJB8 to ameliorate the main mechanism related to mood disorders, starting from the previous findings examined with respect to ameliorating cognitive decline.

## 2. Results

### 2.1. Probiotic Strain Screening in a Dose–Response Study on CaCo-2

**Figure 1:**
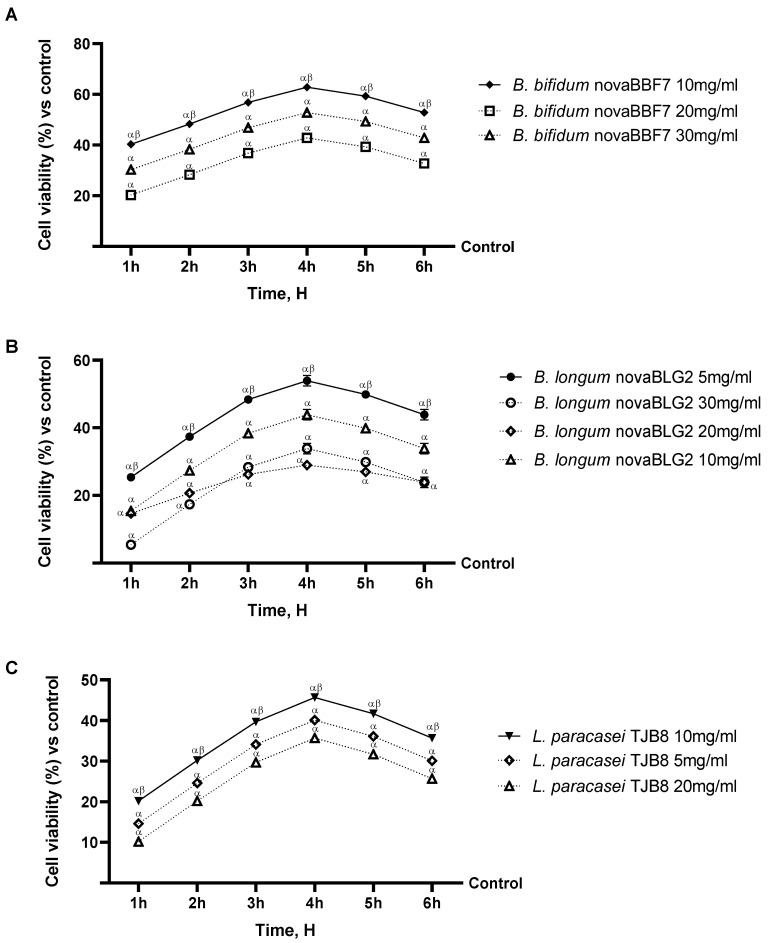
Dose–response study on CaCo-2 cells for assessment of cell viability. In (**A**), cell viability measured by MTT on CaCo-2 after treatment with *B. bifidum* novaBBF7 at different concentrations; in (**B**), cell viability measured by MTT on CaCo-2 after treatment with *B. longum* novaBLG2 at various concentrations; and in (**C**), cell viability measured by MTT on CaCo-2 after treatment with *L. paracasei* TJB8 at different concentrations. Data are expressed as mean ± SD (%) of 5 independent experiments normalized to control to represent a percentage increase in cell viability after treatments compared to the control (0% line). All probiotic strains were *p* < 0.05 vs. control; ^α^ *p* < 0.05 vs. control; ^β^ *p* < 0.05 vs. other concentrations.

An initial dose–response study was conducted to determine the best concentrations of the selected probiotic strains for neuronal experiments. As shown in Figure 1, *B. bifidum* novaBBF7, *B. longum* novaBLG2, and *L. paracasei* TJB8 alone induced a beneficial effect by increasing cell viability compared to the control (*p* < 0.05). In particular, *B. bifidum* novaBBF7 10 mg/mL, *B. Longum* novaBLG2 5 mg/mL, and *L. paracasei* TJB8 10 mg/mL have stronger cell viability effects at 4 h of treatment than other concentrations (*p* < 0.05). Indeed, *B. bifidum* novaBBF7 10 mg/mL increased cell viability by about 19% when compared with the 30 mg/mL concentration and about 47% when compared with the 20 mg/mL concentration; in contrast, *B. longum* novaBLG2 5 mg/mL increased cell viability by about 23%, 59%, and 86% when compared with the 30 mg/mL, 20 mg/mL, and 10 mg/mL concentrations, respectively. Finally, as for *L. paracasei* TJB8, 10 mg/mL is better than the other concentrations because it increases cell viability by about 14% and 14% compared to the 20 mg/mL and 5 mg/mL concentrations. Based on this preliminary study, *B. bifidum* novaBBF7 10 mg/mL, *B. longum* novaBLG2 5 mg/mL, and *L. paracasei* TJB8 10 mg/mL, corresponding to 1.0 × 10^9^ CFU, 0.5 × 10^9^ CFU, and 3.0 × 10^9^ CFU, respectively, were used for subsequent experiments.

### 2.2. Effects of Selected Probiotic Strain on Intestinal In Vitro Model

**Figure 2:**
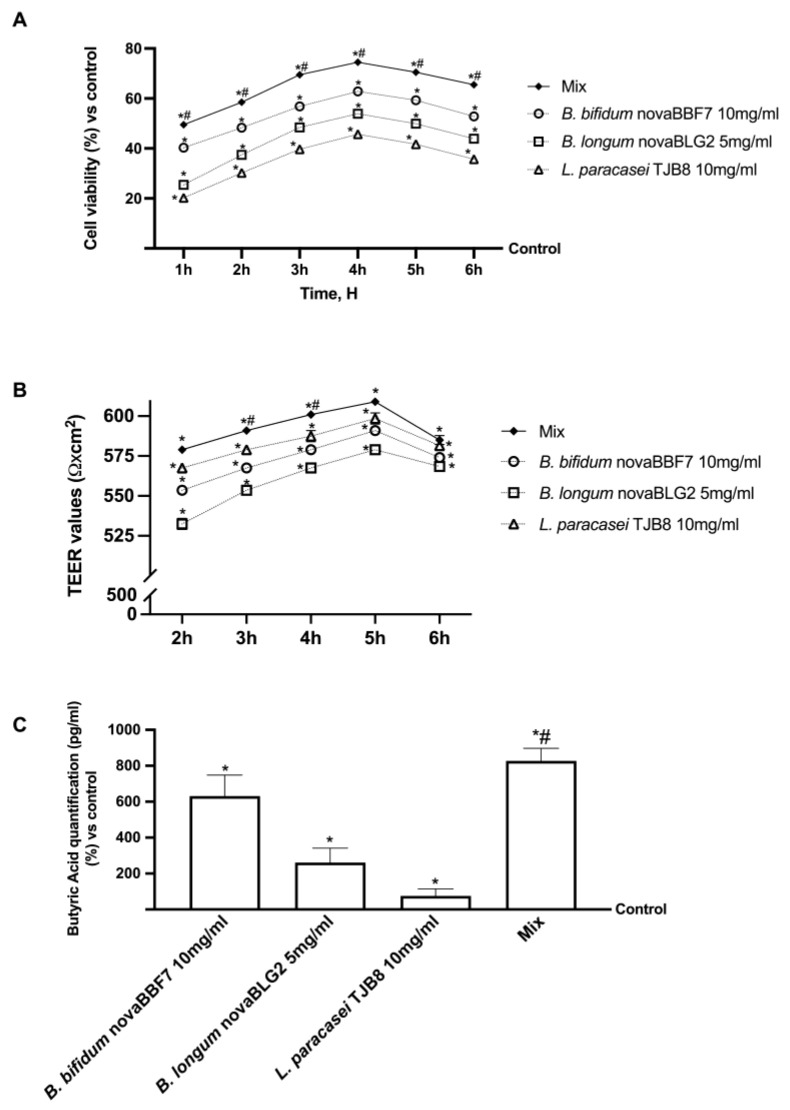
Safety analysis on intestinal in vitro model. In (**A**), cell viability assessed by MTT was evaluated; in (**B**), TEER analysis was performed; and in (**C**), butyric acid quantification assessed by an ELISA Kit was reported. Mix = *B. bifidum* novaBBF7 10 mg/mL + *B. longum* novaBLG2 5 mg/mL + *L. paracasei* TJB8 10 mg/mL. Data are expressed as mean ± SD (%) of 5 independent experiments normalized to control. * *p* < 0.05 vs. control; ^#^ *p* < 0.05 vs. single agents.

Before moving on to brain epithelia, additional experiments were carried out to explore the intestinal effects of probiotics selected in a gut-validated barrier model. Cell viability, transepithelial electrical resistance (TEER) values, and the synthesis of metabolites produced by crossing the intestinal barrier were examined to demonstrate the ability of the selected probiotics to act cooperatively. As shown in Figure 2A, cell viability increases for all tested agents compared to the control (*p* < 0.05); the largest effect was observed at 4 h, with the combination of *B. bifidum* novaBBF7 10 mg/mL, *B. Longum* novaBLG2 5 mg/mL, and *L. paracasei* TJB8 10 mg/mL (named Mix), with an increase in cell viability compared *B. bifidum* novaBBF7 10 mg/mL, *B. Longum* novaBLG2 5 mg/mL, and *L. paracasei* TJB8 10 mg/mL alone (19%, 38%, 63%, respectively, *p* < 0.05). Following this, tests were performed to assess intestinal barrier integrity by evaluating TEER. As shown in Figure 2B, the TEER values of 510 ± 10 Ω·cm^2^ obtained for intestinal CaCo-2 cells appear to agree with the literature results [34]. Notably, Mix had a larger effect on TEER values than individual probiotic strains (*p* < 0.05), implying that the cells form an unbroken monolayer after treatment and preserve adequate intestinal exchange. Finally, butyric acid (Figure 2C) at the basolateral level was examined to establish that the probiotics can produce short-chain fatty acids that cross the intestinal barrier to reach the plasma environment. Specifically, it was noted that Mix was able to amplify the effect of individual agents, resulting in increased absorption across the intestinal barrier; specifically, Mix increases the production of short-chain fatty acids by about 25% compared to *B. bifidum*, about 69% compared to *B. longum* novaBLG2, and about 80% compared to *L. paracasei* TJB8 (*p* < 0.05). Based on these results, it can be hypothesized that Mix and all selected agents can positively influence intestinal permeability without side effects, thus demonstrating the product’s safety.

### 2.3. Biological Effects of Probiotic Metabolites on SHSY-5Y in the Condition of Mood Disorders

**Figure 3:**
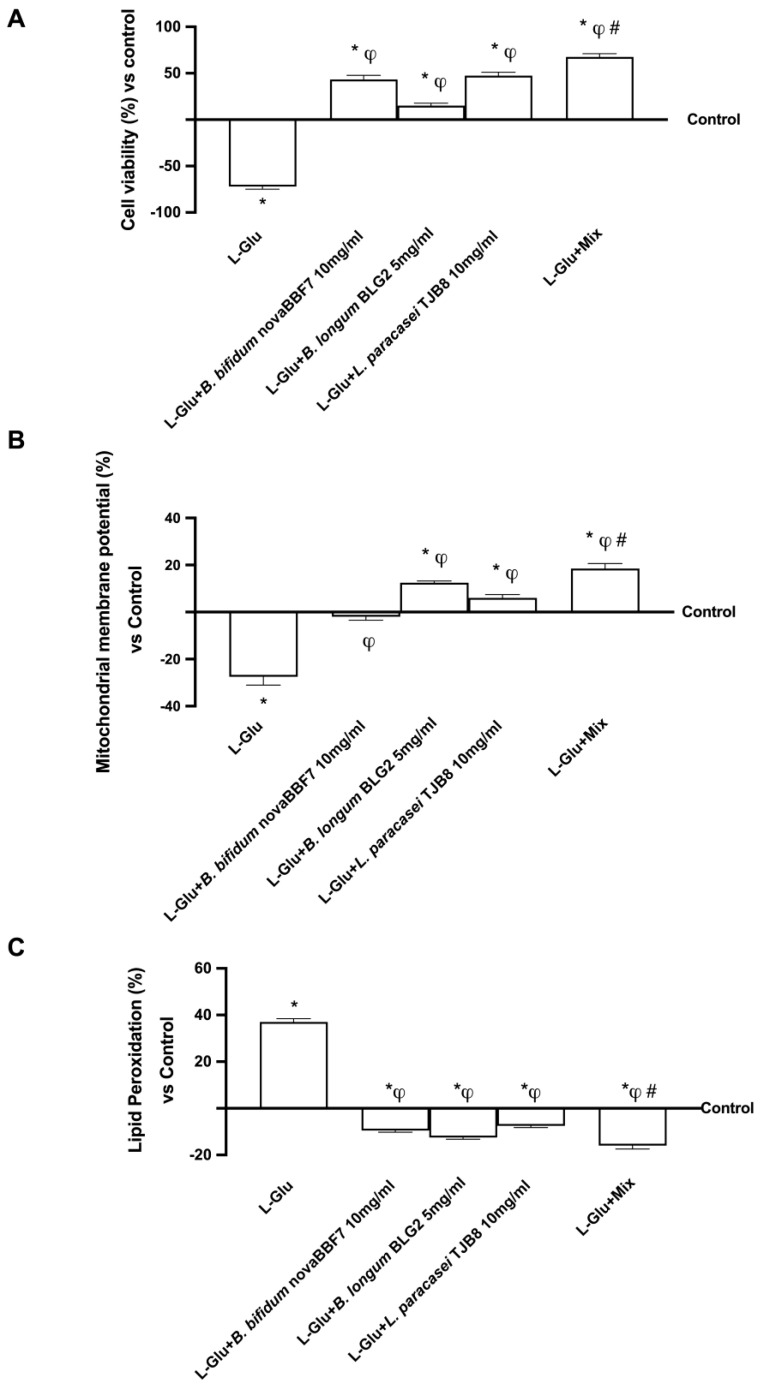
Biological effects of probiotic metabolites on SHSY-5Y in the condition of mood disorders. In (**A**), cell viability was evaluated by the MTT test; in (**B**), mitochondrial membrane potential was analyzed by JC-1 probe; and in (**C**), lipid peroxidation was analyzed by MDA ELISA Kit. L-Glu = L-Glutamate 5 mM; Mix = *B. bifidum* novaBBF7 10 mg/mL + *B. longum* novaBLG2 5 mg/mL + *L. paracasei* TJB8 10 mg/mL. Data are expressed as mean ± SD (%) of 5 independent experiments normalized to control. * *p* < 0.05 vs. control; ^φ^ *p* < 0.05 vs. L-Glu; ^#^ *p* < 0.05 vs. single agents.

Given that glutamate has been shown in the literature to operate as a modulator of mood tones, pretreatment with 5 mM glutamate was used to simulate a condition of depression or, more broadly, mood disorders [35,36]. As shown in Figure 3A, pretreatment with 5 mM L-Glutamic acid (L-Glu) alone significantly reduces cell viability. In contrast, treatment with probiotics alone and in combination can reverse this trend; in particular, as previously reported, the best results are shown by Mix, with a two-fold increase in viability compared to L-Glu alone, about 37% vs. *B. bifidum* novaBBF7, about 78% vs. *B. longum* novaBLG2, and about 17% vs. *L. paracasei* TJB8 (*p* < 0.05). The efficacy of this combined probiotic treatment was also evaluated on the formation of a proton gradient across the inner mitochondrial membrane by JC-1 after pretreatment with 5 mM L-Glu acid (Figure 3B). Again, probiotics alone had a positive effect, inducing a significant increase in JC-1 fluorescence (*p* < 0.05). At the same time, Mix was able to reverse the dissipation of the mitochondrial potential compared with L-Glu pretreatment by two-and-half-fold, shifting the fluorescence signal from green to red, and thus indicating an increase in membrane potential (*p* < 0.05). In addition, Mix also reversed the dissipation of mitochondrial potential compared to individual probiotics by one-fold (*p* < 0.05) vs. *B. bifidum* novaBBF7, about 32% vs. *B. longum* novaBLG2, and about 68% vs. *L. paracasei* TJB8. In addition, lipid damage induced by treatment with L-Glu was also analyzed (Figure 3C); this analysis showed that individual probiotics were able to reduce lipid peroxidation less effectively than Mix; more specifically, Mix reduced peroxidation three-fold compared with L-Glu alone, about 33% compared with *B. bifidum* novaBBF7, about 13% compared with *B. longum* novaBLG2, and about 60% compared with *L. paracasei* TJB8 (*p* < 0.05). These results indicate that the combination of probiotics attenuates L-Glu-induced cell damage by modulating mitochondrial well-being.

**Figure 4:**
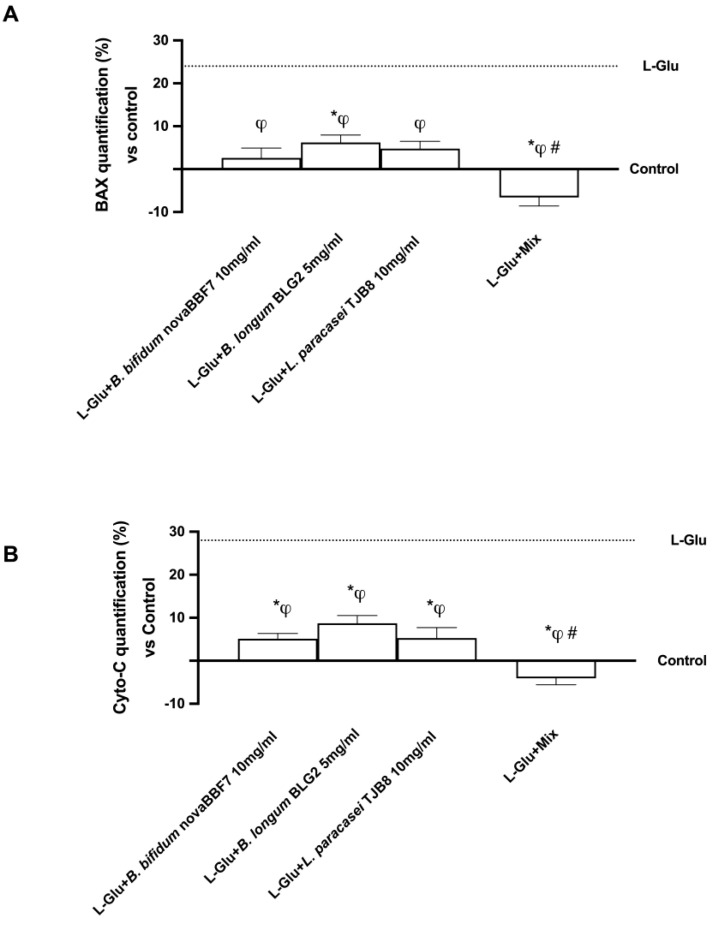
Biological effects of probiotic metabolites on SHSY-5Y in the condition of mood disorders. In (**A**), BAX protein was evaluated by a specific ELISA Kit, and in (**B**), released Cyto-C protein was analyzed by MDA ELISA Kit. L-Glu = L-Glutamate 5 mM (dashed line); Mix = *B. bifidum* novaBBF7 10 mg/mL + *B. longum* novaBLG2 5 mg/mL + *L. paracasei* TJB8 10 mg/mL. Data are expressed as mean ± SD (%) of 5 independent experiments normalized to control. * *p* < 0.05 vs. control; ^φ^ *p* < 0.05 vs. L-Glu; ^#^ *p* < 0.05 vs. single agents.

In addition, the effectiveness of probiotics against apoptosis, both alone and mixed, was also investigated, since anxiety and depression are known to have significant levels of cell death. The levels of BAX, a pro-apoptotic protein, and Cytochrome C, a hallmark of apoptotic process initiation with no chance of shutdown, were then examined. L-Glu pretreatment (dashed line) enhanced both BAX and Cytochrome C (Cyto-C) proteins, respectively, about 24% and 28% compared to control (*p* < 0.05). Still, the probiotic treatment prevented the development of these two apoptotic markers by generating glutamate exposure damage. Individual probiotics and Mix, as shown in Figure 4A, were able to modulate BAX protein, particularly Mix, which was able to reduce BAX protein (*p* < 0.05) by 1.4-fold compared to *B. bifidum* novaBBF7, 1.9-fold compared to *B. longum* novaBLG2, and 1.7-fold compared to *L. paracasei* TJB8, confirming an active role in maintaining neuronal cell homeostasis. MIX also modulated the protein level of Cyto-C, and it was lowered by 2.25-fold compared to *B. bifidum* novaBBF7, 3-fold compared to *B. longum* novaBLG2, and 2.3-fold compared to *L. paracasei* TJB8 (Figure 4B). Together, these findings indicate that MIX can reduce the overexpression of BAX and Cyto-C, consequently blocking the apoptotic process.

**Figure 5:**
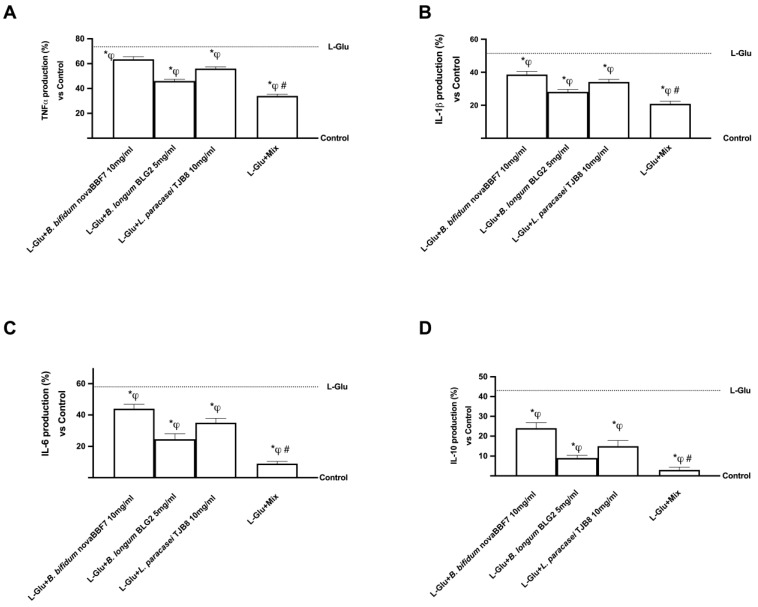
Biological effects of probiotic metabolites on SHSY-5Y in the condition of mood disorders induced by L-Glu (L-Glutamate 5 mM), which is represented in each panel (from A to D) by a dashed line. In (**A**), TNFα was evaluated by a specific ELISA Kit; in (**B**), IL-1β production was analyzed by an ELISA Kit; in (**C**), IL-6 production was evaluated by a specific ELISA Kit; and in (**D**), IL-10 production was analyzed by an ELISA Kit. L-Glu = L-Glutamate 5 mM (dashed line); Mix = *B. bifidum* novaBBF7 10 mg/mL + *B. longum* novaBLG2 5 mg/mL + *L. paracasei* TJB8 10 mg/mL. Data are expressed as mean ± SD (%) of 5 independent experiments normalized to control. * *p* < 0.05 vs. control; ^φ^ *p* < 0.05 vs. L-Glu; ^#^ *p* < 0.05 vs. single agents.

Since major depression is accompanied by significant activation in immune-inflammatory markers, additional experiments were performed to verify the production of some major inflammatory markers involved in the pathophysiology of anxiety and depression. The primary cytokines linked to depression consist of various indicators like Tumor Necrosis Factor α (TNFα), Interleukin (IL)-1β, IL-18, and Interferon (INF)-γ. However, due to the close association of TNFα and IL-1β with the pathophysiological processes of anxiety and depression, these two markers were explicitly investigated. As shown in Figure 5, the exposure to L-Glu (dashed line) improved cytokine production, enhancing the inflammatory process compared to the control (*p* < 0.05). On the contrary, probiotics can ameliorate this condition, reducing the production of inflammatory cytokines, suggesting that this new formulation should maintain its anti-inflammatory ability during depression. The results indicate that the combination of probiotics reverts the L-Glu-induced inflammation; in particular, Mix may reduce the TNFα production by about 46%, 26%, and 39% compared to *B. bifidum* novaBBF7, *L. longum* novaBLG2, and *L. paracasei* TJB8, respectively (*p* < 0.05). In addition, IL-1β production was also analyzed to verify the anti-inflammatory properties of the probiotic combination. Surprisingly, Mix may reduce IL-1β levels by about 45%, 25%, and 39%, respectively (*p* < 0.05). Mix can reduce the production of IL-6 by about 88%, 78%, and 80% compared to *B. bifidum* novaBBF7, *L. longum* novaBLG2, and *L. paracasei* TJB8, respectively (*p* < 0.05). In addition, the Mix reduced IL-10 release by about 84% compared to *B. bifidum* novaBBF7, about 40% compared to *B. longum* novaBLG2, and about 73% compared to *L. paracasei* TJB8 (*p* < 0.05). These data suggest the anti-inflammatory action exerted by the probiotic combination compared with individual strains, suggesting its possible use in managing pathophysiological mechanisms leading to depression.

**Figure 6:**
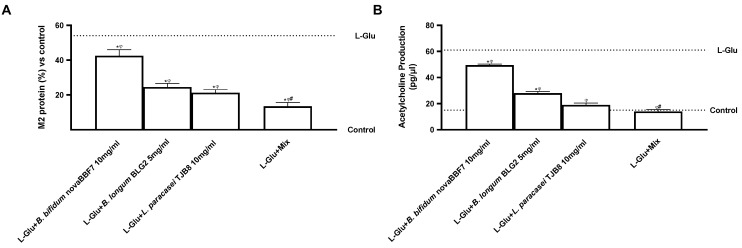
Biological effects of probiotic metabolites on SHSY-5Y in the condition of mood disorders. In (**A**), M2 protein level was evaluated by a specific ELISA Kit; and in (**B**), acetylcholine production was analyzed by an ELISA Kit. L-Glu = L-Glutamate 5 mM (dashed line); Mix = *B. bifidum* novaBBF7 10 mg/mL + *B. longum* novaBLG2 5 mg/mL + *L. paracasei* TJB8 10 mg/mL. Data are expressed as mean ± SD (%) of 5 independent experiments normalized to control. * *p* < 0.05 vs. control; ^φ^ *p* < 0.05 vs. L-Glu; ^#^ *p* < 0.05 vs. single agents.

As a result, decreased expression of these receptors may enhance Ach release, and elevated central choline levels have been seen in depressed patients. Finally, the involvement of Muscarinic Acetylcholine Receptor (M2)-AchR levels is explored. M2-AchR protein level and Ach production increased after L-Glu administration, as seen in Figure 6, indicating its effects in generating neuronal injury (*p* < 0.05). On the other hand, probiotic treatment was able to reverse this condition, decreasing both muscarinic receptor level and Ach production when compared to L-Glu (*p* < 0.05). However, the maximum effect was observed with Mix (*p* < 0.05), confirming the cooperative role of these probiotics in modulating the cholinergic system to act in mood mechanisms. Mix reduced the M2 receptor by about 64%, 32%, and 25% compared to *B. bifidum* novaBBF7, *L. longum* novaBLG2, and *L. paracasei* TJB8, respectively (*p* < 0.05). Mix was also able to lower acetylcholine production by about 72% when compared to *B. bifidum* novaBBF7, about 48% when compared to *B. longum* novaBLG2, and about 40% when compared to *L. paracasei* TJB8 (*p* < 0.05). These findings suggest that the combination of probiotics operates on the cholinergic system by correcting the in vitro processes behind L-Glu-induced mood disorders via cooperative actions.

**Figure 7:**
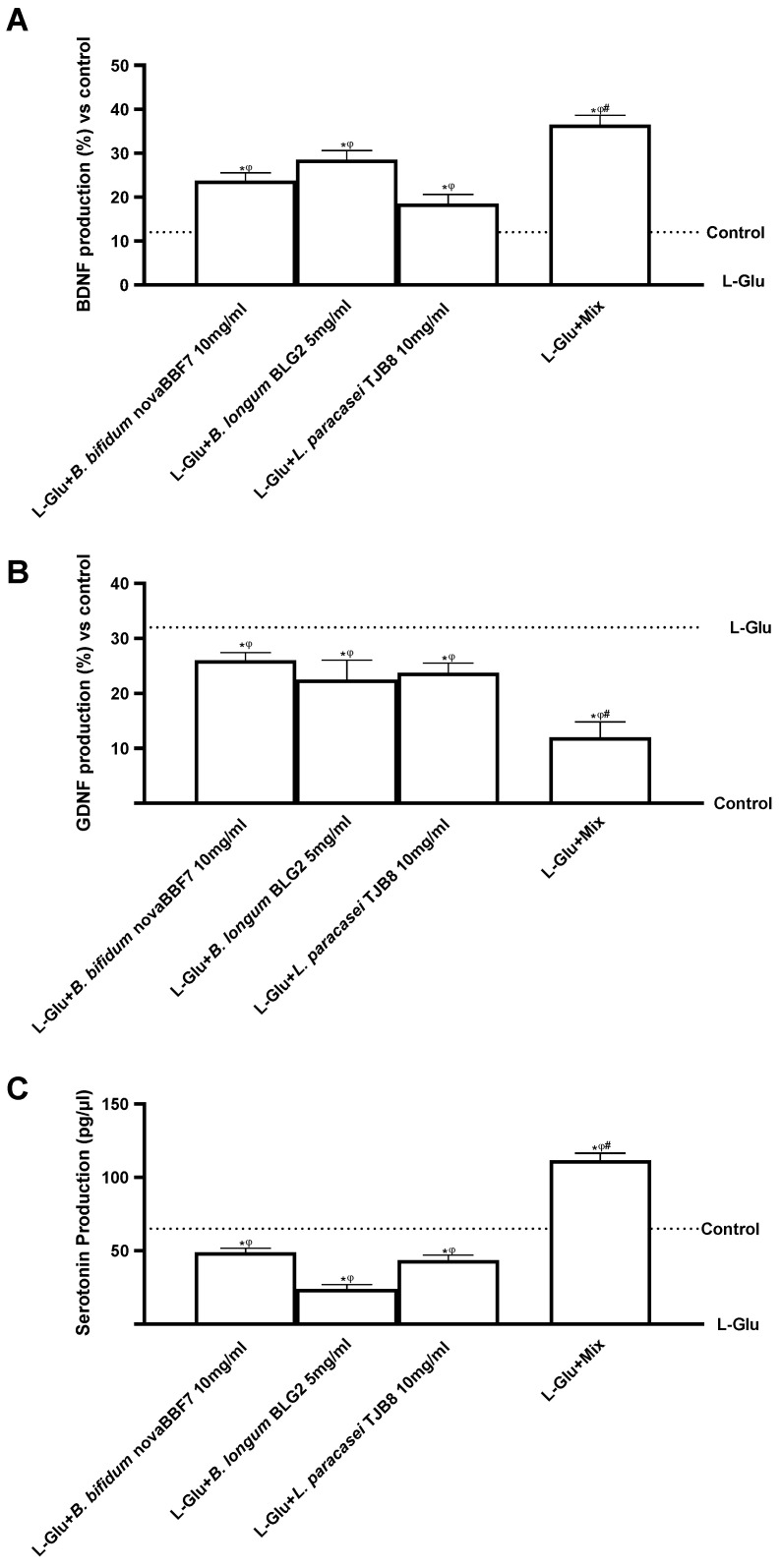
Biological effects of probiotic metabolites on SHSY-5Y in the condition of mood disorders. In (**A**), BDNF quantification was evaluated by an ELISA Kit; in (**B**), GDNF quantification was analyzed by an ELISA test; and in (**C**), serotonin production was analyzed by an ELISA Kit. L-Glu = L-Glutamate 5 mM (dashed line); Mix = *B. bifidum* novaBBF7 10 mg/mL + *B. longum* novaBLG2 5 mg/mL + *L. paracasei* TJB8 10 mg/mL. Data are expressed as mean ± SD (%) of 5 independent experiments normalized to control. * *p* < 0.05 vs. control; ^φ^ *p* < 0.05 vs. L-Glu; ^#^ *p* < 0.05 vs. single agents.

Finally, brain-derived neurotrophic factor (BDNF), glial cell line-derived neurotrophic factor (GDNF), and serotonin productions were analyzed. As shown in Figure 7, GDNF/BDNF productions were significantly increased under L-Glu-treated samples compared to the control (*p* < 0.05). Nevertheless, Mix restored the damage by activating the survival pathway and maintaining the balance between BDNF and GDNF levels better than the single agents during L-Glu exposure (*p* < 0.05). Specifically, Mix increased BDNF by about 37% compared with *B. bifidum* novaBBF7, about 20% compared with *B. longum* novaBLG2, and about 48% compared to *L. paracasei* TJB8 (*p* < 0.05). Similarly, Mix reduced about 52%, 41%, and 43% of the GDNF compared to *B. bifidum* novaBBF7, *L. longum* novaBLG2, and *L. paracasei* TJB8, respectively (*p* < 0.05). Finally, Mix increased serotonin production by about 60%, 79%, and 65% compared to *B. bifidum* novaBBF7, *L. longum* novaBLG2, and *L. paracasei* TJB8, respectively. These results are confirmed by the induction of serotonin by Mix (*p* < 0.05), the production of which plays a key role in the pathophysiology of stress, triggering impairment of synaptic plasticity, neuronal dysfunction, and neuroinflammation in vitro.

## 3. Discussion

Two clusters of genes responsible for the formation of synapses between neurons in the brain and neurons in the gastrointestinal (GI) tract exhibit similarities, and any genetic mutation has the potential to cause abnormalities in both the brain and the GI tract [1,2]. In more recent times, studies have shown a strong correlation between psychiatric conditions such as anxiety and depression and bowel problems, mainly due to an inflammatory network [37,38,39,40,41]; in particular, the genetic overlap between irritable bowel syndrome (IBS) and mood and anxiety disorders and the lack of signals implicating genes expressed specifically in the gut or overlapping with other intestinal disorders were relevant. There has been a growing concern regarding the correlation between neural connections in the brain and the gastrointestinal tract in relation to mood disorders [42]. Additionally, it is known that the gut and the brain can communicate through several systems, such as the immune system, the metabolism of tryptophan, the vagus nerve, and the enteric nervous system, which uses peptidoglycans, branched-chain amino acids, and short-chain fatty acids produced by microbes that act as neurotransmitters. This turns out to be critical for various physiological activities, including cognitive ones. In addition, when levels of depressive symptoms reach clinically meaningful thresholds, they become associated with an increased risk of mild cognitive impairment and dementia, with high risk; prospective studies in middle-aged adults also indicate that clinically meaningful anxiety levels are associated with an increased risk of dementia diagnosis [33]. As a result, the significance of these connections in the field of studying the molecular and physiological underpinnings of mental and neurodegenerative illnesses is growing [3]. Furthermore, a recent study has demonstrated the causative impact of GMB on human behavior and brain development and function, as well as an understanding of the underlying molecular pathways. GMB, for example, has been connected to immune cell proliferation and function in the CNS, particularly microglia [4,5,6]. Butyrate, 5HT, norepinephrine, dopamine, and aminobutyric acid are only a few of the specific chemicals the GMB can produce on its own or in conjunction with other organs [11]. Analysis of the microbiota–gut–brain axis and the effects that bacteria in the microbiota may have on the functioning of the CNS has shown that, for psychiatric disorders, an exciting approach is probiotic supplementation [12]. Indeed, in humans, probiotics can lower proinflammatory cytokines and oxidative stress and alleviate symptoms of depression and anxiety [13]. The present study aimed to investigate the role of various probiotics and the effects of their metabolites in improving a condition of anxiety and depression. Based on information regarding the interaction between the microbiome and the brain and the role of the microbiome in the development of mood disorders, scientific research has focused in recent years on validating the use of probiotics for the benefit of psychiatric disorders, as evidenced by the increase in clinical evidence. Since the complex human internal environment remains difficult to create in an in vitro model, this unique model (the gut–brain axis) simulates signaling from the intestinal lumen to nerves while replicating the characteristics of the tight junctional barrier in the large intestine. For this purpose, *B. bifidum* novaBBF7, *B. longum* novaBLG2, and *L. paracasei* TJB8, alone and combined, were tested on intestinal cells to evaluate their safety and efficacy. These probiotics showed greater beneficial effects than the others. This suggested their possible use in combination to amplify the beneficial effect not only at the gut level but also at the brain level. It is apparent from the data obtained at the intestinal level that the combined probiotic treatment can increase cell viability, maintain the integrity of the intestinal cell monolayer, and increase the production of short-chain fatty acids (SCFAs), such as butyric acid, which crosses the intestinal barrier, reaching the bloodstream to act as a second messenger in the various body districts. Once the SCFAs reach the brain level, they can increase mitochondrial metabolism, reverse membrane potential dissipation, and prevent lipid peroxidation that arises during the pathophysiological processes of altered mood tones. On a molecular level, it was also found that individual probiotics and their combination could control the development of two apoptotic-associated indicators, BAX and Cyto-C, related to damage caused by glutamate exposure in the nervous system. As a result, we verified that the chosen probiotics had a more than favorable effect on preserving cell viability and survival in an in vitro model of an injured nerve. In addition, as the results described above suggested, the individual probiotics and their combination showed an even more pronounced anti-inflammatory effect by reducing the production of the main inflammatory markers that normally occur in depressive states, such as TNFα, IL-1β, IL-6, IL-10, IL-18, and INF-γ [43,44]. Indeed, the reduction of cytokine levels after stimulation with Mix was increased by about 80% over individual probiotic strains. It is important to remember that the “cytokine theory of depression” proposes that enhanced production of proinflammatory cytokines (such as IL-6, IL-1β, IFN-γ, and TNFα) is associated with the pathogenesis of depression [45]. Although most studies on the “cytokine theory of depression” are centered on increased levels of proinflammatory cytokines, the role of anti-inflammatory IL-10, one of the most important anti-inflammatory cytokines, was investigated in depression [46]. IL-10 impacts several symptoms associated with depression, namely, helplessness, sleep disturbances, and pain perception [45,47]. Another important cytokine, IL-6, is often induced together with the proinflammatory cytokines, such as TNFα, in many alarm conditions, and circulating IL-6 plays an important role in the induction of acute phase reactions. IL-6 is a pleiotropic cytokine with pro- and anti-inflammatory properties [48]. Our results align with the latest scientific evidence showing how antidepressants can reduce pro-inflammatory cytokines (TNFα, IL-1β, and IL-6) and the anti-inflammatory cytokine IL-10 [43,49].

Nerve factors may be gut- or brain-derived, and they play important roles in systemic homeostasis and regulating neural circuit development and plasticity. Specifically, BDNF and GDNF co-modulate each other in their physiological responsibilities of controlling neuronal circuit formation and plasticity. Serotonin increases neurogenesis and neuronal survival in reaction to serotonin, and BDNF/GDNF promotes neurogenesis and neuronal survival in response to serotonin. The pathophysiology of depression has been linked to disruptions in the serotonin–BDNF signaling pathway. This system affects neuron formation, survival, and differentiation, and it acts synergistically on the survival of injured motor neurons during depressive episodes. Moreover, major depression is associated with substantial activation of immune-inflammatory markers, specifically proinflammatory cytokines, which can disrupt the brain’s regulatory and signaling mechanisms related to behavioral and emotional functions. Finally, multiple studies have found lower M2-AchR levels in patients with bipolar illness and major depressive disorder, demonstrating that the cholinergic system is involved in mood regulation. In addition, after probiotic treatment, levels of pro-inflammatory cytokines, M2 receptor activity, and acetylcholine production previously increased by L-Glu-induced damage were also reduced. Ultimately, the probiotic combination treatment restored BDNF activity, maintaining the relationship with GDNF and increasing the levels of serotonin produced after damage by L-Glu. 

## 4. Materials and Methods

### 4.1. Substances Preparation

*Bifidobacterium longum* novaBLG2 (DSM 34339) (named *B. longum* novaBLG2), *Bifidobacterium bifidum* novaBBF7 (DSM 34336) (named *B. bifidum* novaBBF7), and *Lacticaseibacillus paracasei* TJB8 (DSM 33129) (named *L. paracasei* TJB8) donated by Probionova SA (Lugano, Switzerland) were prepared at the moment of use. In particular, before performing each stimulation, a different pack of the product was used by reconstituting and mixing probiotics into DMEM without red phenol (Merck Life Science, Rome, Italy), supplemented with 0% FBS and 2 mM L-glutamine solution (Merck Life Science, Rome, Italy). For each set of experiments, the samples were diluted in a culture medium immediately before use to reach the desired final concentration. Different concentrations of the individual agents were tested in a dose–response study. These in vitro concentrations are equivalent to a probiotic daily consumption ranging from 0.5 to 3.0 × 10^9^ CFU. The most effective dose identified for each strain was evaluated in subsequent experiments for both the individuals and their combination. The dosages tested are the same as those used in a previous study to explore the ability of the same strains to modulate cognitive decline. This choice depends on the several findings reported in the literature about the role of the same probiotics in ameliorating both cognitive decline and mood disorders.

### 4.2. Cell Cultures

As an experimental model, the human epithelial intestinal CaCo-2 cell line obtained from ATCC (Manassas, VA, USA) was utilized to forecast intestinal absorption characteristics after oral ingestion [50]. This cell line was grown in Advanced Dulbecco’s Modified Eagle’s Medium/Nutrient F-12 Ham (Adv DMEM-F12; GIBCO^®^ ThermoFisher Scientific, Waltham, MA, USA), which contained 1% penicillin-streptomycin (Merck Life Science, Rome, Italy), 10% fetal bovine serum (FBS, Merck Life Science, Rome, Italy), and 2 mM L-glutamine. The culture was kept in an incubator at 37 °C and 5% CO_2_ [51]. To preserve paracellular permeability and transport, experiments used cells at passage numbers 26–32 [52]. Cells were seeded 1 × 10^4^ in 96-well plates after 80% confluence to make an MTT-based In Vitro Toxicology Assay Kit (Merck Life Science, Rome, Italy) after synchronizing them with Adv DMEM without red phenol at 37 °C for eight hours with 0.5% FBS (GIBCO^®^ ThermoFisher Scientific, Waltham, MA, USA), 2 mM L-glutamine, and 1% penicillin-streptomycin (Merck Life Science, Rome, Italy). Additionally, 2 × 10^4^ cells were plated on a 6.5 mm Transwell^®^ (Corning^®^ Costar^®^, Merck Life Science, Rome, Italy) with a 0.4 μm pore polycarbonate membrane insert in a 24-well plate for absorbance analysis [53], maintaining them in a complete medium, by changing every other day on the basolateral and apical sides for 21 days [54]. To match the small intestine lumen pH, the medium was pH 6.5 on the apical side before stimulation. However, basolateral pH 7.4 represented blood [55]. This in vitro model is widely used [53] and approved by the EMA and FDA to predict human oral absorption, metabolism, and bioavailability [56,57].

SH-SY5Y cells from ATCC (Manassas, VA, USA) were cultured in a 1:1 mixture of Advanced Dulbecco’s Modified Eagle Medium F12 (Adv DMEM F12; GIBCO^®^ ThermoFisher Scientific, Waltham, MA, USA) and Adv DMEM supplemented with 10% FBS, and cells were incubated at 37 °C with 5% CO_2_ and 95% humidity [58]. The investigations utilized cells with passage numbers ranging from 3 to 20. The cells were cultured using various methods: Cell viability, mitochondrial membrane metabolism, and lipid peroxidation assays were performed using specific kits in 96-well plates with 1 × 10^4^ cells. Apoptotic intracellular pathways, including IL-6 and IL-10 production, M2 detection, BDNF and GDNF, and serotonin production were investigated using an ELISA Kit with 4 × 10^5^ cells in 6-well plates.

### 4.3. Experimental Protocol

Two sets of tests were conducted. The first group looked at how probiotics could modify the intestinal barrier’s functioning without any negative side effects. On the other hand, the second study used the gut–brain axis model to study the impact of probiotic metabolites on gut cells. It evaluated the intracellular pathways that underlie the modulation of anxiety and depression in neural cells. Using the CaCo-2 cell line, the first investigation examined cell viability in a dose–response study using the MTT assay to rule out the harmful effects of *B. longum* novaBLG2, *B. bifidum* novaBBF7, and *L. paracasei* TJB8 independently and in combination [59]. Then, probiotic strains alone and combined were tested on a 3D intestinal in vitro barrier model to establish cell survival using the MTT test and intestinal stability using TEER analysis, indicating proper epithelial integrity maintenance. Finally, butyric acid was measured using an ELISA assay to confirm the role of one SCFA in cell signaling control throughout the entire body. In all of these tests, the cells were given time-dependent treatments ranging from 1 to 6 h [51]. In the second step of the study, a model of the gut–brain axis was developed to study the effects of probiotic strains in modulating the anxiety and depression condition determined by Glutamate 5 mM [35]. Neuronal cells were treated with the basolateral media of the intestinal barrier for 24 h to imitate the correct treatment dosage. Neuronal cells were treated with the basolateral media of the intestinal barrier for 24 h to mimic the appropriate treatment dosage. At the end of stimulation, mitochondrial metabolism, mitochondrial membrane potential, and lipid peroxidation were investigated; in addition, apoptotic molecular pathway activity, including BAX and Cyto-C proteins and TNFα, IL-1β, IL-6, and IL-10 production, were examined by ELISA Kit. Finally, M2 detection, acetylcholine production, BDNF production, GDNF production, and serotonin production were examined using a specific ELISA kit.

### 4.4. Gut–Brain Axis

The co-culture approach using CaCo-2 and SHSY-5Y cell lines on Transwell^®^ plates was conducted using a standard protocol outlined in the literature [60]. A semipermeable membrane with a pore size of 0.4 μm (Corning^®^ Costar^®^, Merck Life Science, Rome, Italy) was used to separate the two chambers filled with Adv DMEM medium (GIBCO^®^ Thermo Fisher Scientific, Waltham, MA, USA). The insert co-culture model is designed as follows: CaCo-2 cells were grown in dense layers on filter inserts (25,000 cells per insert). On the seventh development day, low-density SH-SY5Y cells were plated in a separate 24-well (400 SH-SY5Y cells/well) flat-bottom plate. These neuroblastoma-sized neurites were detected when cells were plated at n = 400 cells/well and left untreated for 5 days. Instead, stellate structures appear within 24 h of development when plating n = 25,000 cells/well. This happens because SH-SY5Y cells help each other grow.

The cells on the culture media will have developed a high TEER value (≥500 Ω·cm^2^) 14 days after intestinal epithelium maturation, suggesting tight junction formation. Five more days were spent growing each of the two cell lines independently. After that, the two cell lines were kept in the incubator for 15 h. When the two lines were mixed before stimulation, TEER was re-evaluated to guard against any alteration of the intestinal cell monolayer. The cells were subjected to cell viability tests, quantification of ROS production, and evaluation of mitochondrial metabolism during the brain degenerative process.

### 4.5. Cell Viability Test

According to the literature [61], an MTT-based In Vitro Toxicology Assay Kit (Merck Life Science, Rome, Italy) was used on a 96-well plate to measure cell viability. The cells were cultured with 1% MTT dye for 2 h at 37 °C in an incubator with 5% CO_2_, 95% humidity after each stimulation. Crystals were dissolved in MTT Solubilization Solution in equal amounts. A spectrometer (Infinite 200 Pro MPlex, Tecan, Männedorf, Switzerland) measured absorbance at 570 nm with correction at 690 nm to determine cell viability. The data were expressed as an increased percentage normalized to the control value (0%) to explain the percentage increase or decrease in the effects of the substances tested and between the groups of treatments.

### 4.6. Intestinal Integrity Analysis

CaCo-2 cells were plated on inserts and monitored using EVOM3 (World Precision Instruments, Sarasota, FL, USA) on alternate days for 21 days. Experiments began when TEER reached ≥500 Ω·cm^2^. The literature recommends TEER values of ≥500 ± 52.9 Ω·cm^2^ for transport studies [51].

### 4.7. Butyric Acid Quantification

The butyric acid generated by stimulating CaCo-2 cells with probiotics was measured using an ELISA Kit from Cloud-Clone in Wuhan, China, following the manufacturer’s guidelines [62]. Each sample’s absorbance was measured at 450 nm after adding the stop solution using a plate reader (Infinite 200 Pro MPlex, Tecan, Männedorf, Switzerland). The data were expressed as mean (pg/mL) compared to control (0% line) by interpolarizing the OD using a standard curve that ranged from 10,000 pg/mL to pg/mL.

### 4.8. Oxygen Consumption and Mitochondrial Membrane Potential

According to the literature, an Oxygen Consumption/Mitomembrane Potential Dual Assay Kit (Cayman Chemical Company, Ann Arbour, MI, USA) was used to simultaneously test oxygen consumption and mitochondrial membrane potential [63]. Briefly, 100 µL of JC-1 Staining Solution was added, and the plate was incubated for 15 min at 37 °C. Then, the plate was centrifugated at room temperature for 5 min at 400× *g*. After removing the supernatant, 200 µL of assay buffer was added, and the plate was centrifugated. These two passages were repeated. Finally, 100 µL of assay buffer was added. Healthy cells with mainly JC-1 J-aggregates can be measured by excitation and emission wavelengths at 540 and 570 nm, respectively. In comparison, a fluorescence spectrometer can detect apoptotic or unhealthy cells with mainly JC-1 monomers at an excitation/emission of 485/535 nm (Infinite 200 Pro MPlex, Tecan, Männedorf, Switzerland). The results are expressed as mean ± SD (%) compared to control cells (0% line).

### 4.9. Lipid Peroxydation Assay Kit

The TBARS Assay Kit (Cayman Chemical, Tallinn, Estonia) measured cell lipid peroxidation [64], adding 100 μL of SDS solution to 100 μL of sample or standard. Before boiling for 1 h, each vial received 4 mL of dye reagent. Post-cooling, samples were centrifuged at 1600× *g* for 10 min at 4 °C. A total of 150 μL of samples or standard was added to 96 multi-wells, and absorbance was measured at 530–540 nm using a spectrometer (Infinite 200 Pro MPlex, Tecan, Männedorf, Switzerland). Comparing concentration to a standard curve (0–50 µM), findings are provided as mean ± SD (%) compared to untreated cells (0% line).

### 4.10. BAX ELISA Assay

According to the manufacturer’s instructions, the BAX ELISA Kit (MyBioSource, San Diego, CA, USA) was used to analyze BAX protein in SHSY-5Y cell lysates [65]. A total of 100 μL of sample or standard was added to each well, followed by incubation at 37 °C for 90 min. To each well, 100 μL of biotinylated antibody was added, followed by incubation for 60 min at 37 °C and cleaning, and then 100 μL of SABC working solution was added for 30 min at 37 °C. Next, 90 μL of TMB substrate was added, followed by incubation at 37 °C for 15–30 min. The samples were analyzed at 450 nm by a Tecan Infinite 200 Pro MPlex spectrometer (Männedorf, Switzerland). Results are reported as pg/mL compared to a standard curve (0–2000 pg/mL) and as mean ± SD (%) compared to control cells (0% line).

### 4.11. Cytochrome C Assay Kit

The Cytochrome-C ELISA Kit (MyBiosource, San Diego, CA, USA) was used to measure the amount of cytochrome C in SHSY-5Y cell lysates, following the manufacturer’s instructions [66]. At each 100 μL of each sample, an equal volume of 100 μL of detection solution A was added, followed by incubation for 45 min at 37 °C. Then, at the wells, after washing, 100 μL of detection solution B was added to incubating wells for 45 min at 37 °C. The plate was incubated at 37 °C for 20 min in the dark after adding 90 μL of substrate solution to each well. A total of 50 μL stop solution was employed to stop the reaction. The absorbance was analyzed with a spectrometer (Infinite 200 Pro MPlex, Tecan, Männedorf, Switzerland) at 450 nm, and the concentration was quantified in ng/mL by comparing the data to the standard curve (15.6–500 nmol/L). The data are presented as mean ± SD (%) versus control cells (0% line).

### 4.12. TNFα Assay Kit

According to the guidelines provided by the manufacturer, an analysis of TNFα production on the SHSY-5Y supernatant under conditions of oxidative stress was conducted utilizing the Human Tumor Necrosis Factor α ELISA Kit (Merck Life Science, Milan, Italy). Subsequently, 100 µL of the sample was dispensed into individual wells of a 96-well ELISA plate, followed by an incubation period at room temperature lasting 2 h, then overnight at 4 °C. This was succeeded by five consecutive washes using a washing buffer post-incubation and adding 100 µL of biotinylated anti-TNFα into each well. Following a 2 h incubation at room temperature, the contents of each well were aspirated and washed five times before the introduction of 100 µL Streptavidin–HRP for a 1 h incubation. Subsequently, the plate with Streptavidin–HRP solution, 100 µL of chromogen solution, was incubated in each well for 30 min at room temperature without light. The absorbance of each well was then determined at 450 nm utilizing a Tecan plate reader after applying stop solution [67]. The data are presented as mean ± SD (%) relative to the untreated control sample (0% line).

### 4.13. IL-1β Assay Kit

IL-1β in SHSY-5Y lysates was quantified following the manufacturer’s instructions using an IL-1β ELISA Kit (R&D Systems, Minneapolis, MN, USA). The procedure involved adding IL-1β-containing culture medium to a microplate strip (100 μL/well), incubating for 2 h at room temperature, mixing with IL-1β conjugate, and further incubating for another 2 h at room temperature. Thorough washing steps were performed before and after the two incubations. Subsequently, 100 μL of substrate solution was added to generate chemiluminescence, and the absorbance was measured using a microplate reader at 450 nm with correction at 570 nm. IL-1β quantification compared the sample readings to the standard curve generated [68].

### 4.14. Human IL-6 (Interleukin-6) ELISA Kit

The Human IL-6 (Interleukin-6) ELISA Kit (FineTest, Wuhan, China) was applied to verify the IL-6 production according to the instructions [69]. After adding 100 µL of each sample to each well, the plate was incubated at 37 °C for 90 min. After incubation, each well was cleaned twice with a wash buffer. The plate was incubated at 37 °C for 60 min after adding 100 µL of biotin-labeled antibody working solution to the wells. After incubation, each well was washed three times with wash buffer to remove the solution. After adding 100 µL of HRP-Streptavidin Conjugate to each well, the plate was incubated at 37 °C for 30 min. After washing the wells five times, 90 µL of TMB substrate was added to each well. After 10–20 min, 50 µL of stop solution was added to each well, and the plate was read at 450 nm using a plate reader (Infinite 200 Pro MPlex, Tecan, Männedorf, Switzerland). A curve was drawn to correlate color intensity (OD) with standard concentration (4.688–300 pg/mL). Results were presented as mean ± SD (%) vs. control (0% line).

### 4.15. Interleukin-10 ELISA Kit

Following instructions, an ELISA Kit (FineTest, Wuhan, China) measured Interleukin-10. The plate was incubated at 37 °C for 90 min after adding 100 µL of each sample to each well. After incubation, waste was collected from each well, and the plate was washed twice with a wash buffer. The plate was incubated at 37 °C for 60 min after adding 100 µL of biotin-labeled antibody working solution to the wells. The wells were rinsed three times with wash buffer after incubation to remove the solution. The plate was incubated at 37 °C for 30 min after adding 100 µL of HRP-Streptavidin Conjugate to each well. Finally, each well was rinsed five times, and 90 µL of TMB substrate was added. A plate reader (Infinite 200 Pro MPlex, Tecan, Männedorf, Switzerland) was used to read the plate at 450 nm after adding 50 µL of stop solution to each well after 10–20 min. A standard curve relates O.D. intensity to standard concentration (7.813–500 pg/mL). The data are presented as mean ± SD (%) vs. control cells (0% line).

### 4.16. Human Muscarinic Acetylcholine Receptor M2/CHRM2 ELISA Kit

According to the manufacturer’s instructions, M2 protein was detected using a Human Muscarinic Acetylcholine Receptor M2/CHRM2 ELISA Kit (Colorimetric) (Novus Biologicals, San Diego, CA, USA). Briefly, 100 µL of each standard or sample was added to each well, and the plate was incubated for 90 min at 37 °C. Then, the liquid was removed, and 100 µL of Biotinylated Detection Ab was added. After 1 h at 37 °C, the liquid was removed, and the plate was washed 3 times before adding 100 µL of HRP Conjugate for 30 min at 37 °C. After 5 washes, 90 µL of substrate reagent was added, and the plate was incubated for 15 min at 37 °C. Finally, 50 µL of stop solution was added, the samples were analyzed with a spectrometer (Infinite 200 Pro MPlex, Tecan, Männedorf, Switzerland) at 450 nm, and the concentration was expressed as ng/mL against a standard curve (range 0.16 ng/mL to 10 ng/mL). The results are expressed as means ± SD (%) compared to control cells (0% line).

### 4.17. Choline/Ach Quantification Kit

A Choline/Ach Quantification Kit (Merck Life Science, Rome, Italy) was used to determine the Ach level, as reported in the literature [70], following the manufacturer’s instructions. Insoluble material was removed by rapidly homogenizing SHSY-5Y cells in 4 volumes of cold choline assay buffer and centrifuging at 13,000× *g* for 10 min at 4 °C. A horizontal shaker was used to mix 50 mL of the reaction Mix with 50 mL of each sample in 96-well plates for 30 min at room temperature and in the dark. The concentration of Ach was determined by comparing the absorbance at 570 nm to choline standards (0–5 nmol) using a spectrometer (Infinite 200 Pro MPlex, Tecan, Männedorf, Switzerland). Results are presented as means ± SD (%) compared to control cells (0% line).

### 4.18. BDNF ELISA Kit

Following manufacturer guidelines, a Rat BDNF Elisa Kit (Thermo Scientific^TM^, Waltham, MA, USA) quantified brain-derived neurotrophic factor (BDNF) [71]. Briefly, 70 μL of standard and 70 μL of the sample were mixed in 96-well plates on a horizontal shaker for 2.5 h at room temperature, away from light. After thoroughly washing the plate with 1× wash buffer, it was allowed to dry upside down on a paper towel. Subsequently, 70 μL of 1× prepared biotinylated detection antibody was added to each well, followed by incubation for 45 min at room temperature with gentle shaking. After 45 min of HRP-conjugated streptavidin incubation, 66.7 μL of TMB substrate solution was added for 30 min, followed by 33.7 μL of stop solution to stop the reaction. A spectrometer (Infinite 200 Pro MPlex, Tecan, Männedorf, Switzerland) measured absorbance at 450 nm and computed BDNF concentration by comparing findings to the standard curve. Results are presented as mean ± SD (%) vs. control cells (0% line).

### 4.19. GDNF ELISA Kit

GDNF production was measured using an ELISA Kit (MyBioSource, Cambridge, UK) according to the instructions provided by the manufacturer [72]. Briefly, 100 µL of each standard or sample was added to each well, and the plate was incubated for 90 min at 37 °C. Subsequently, 100 µL of biotin conjugate anti-Human GDNF Ab was added, followed by three washes at 37 °C, before adding 100 µL of ABC working solution for 30 min. A total of 90 µL of Color Developing Reagent was combined with the plate, followed by incubation at 37 °C for 15 min. Finally, 100 µL of stop solution was added. The samples were analyzed with a spectrometer (Infinite 200 Pro MPlex, Tecan, Männedorf, Switzerland) at 450 nm. The concentration is expressed as ng/mL against a standard curve (range 31.2 pg/mL to 2000 pg/mL), and the results are expressed as means ± SD (%) compared to control cells (0% line).

### 4.20. Human HTR1A (5-Hydroxytryptamine Receptor 1A) ELISA Kit

As instructed, HTR1A concentration was measured using the Human HTR1A (5-hydroxytryptamine receptor 1A) ELISA Kit (FineTest, Wuhan, China). After adding 100 μL of each sample to each well, the plate was incubated at 37 °C for 90 min. After incubation, each well was cleaned twice with a wash buffer. The plate was incubated at 37 °C for 60 min after adding 100 μL of biotin-labeled antibody working solution to the wells. After incubation, each well was washed three times with wash buffer to remove the solution. After adding 100 µL of SABC working solution to each well, the plate was incubated at 37 °C for 30 min. After washing the wells five times, 90 µL of TMB substrate was added to each well. After 10–20 min, 50 µL stop solution was added to each well, and the plate was read at 450 nm using a plate reader (Infinite 200 Pro MPlex, Tecan, Männedorf, Switzerland). A standard curve is plotted connecting O.D. intensity to standard concentration (0.156 to 10 ng/mL). Measurements are expressed as mean ± SD (%) vs. control cells (0% line) [73].

### 4.21. Statistical Analysis

The results are shown as mean ± SD of at least 5 biological replicates, with each replication performed 3 times per protocol. Group comparisons were performed using one-way ANOVA with Bonferroni’s post hoc test or Mann–Whitney’s U test in GraphPad Prism 10.2.2 (GraphPad Software, La Jolla, CA, USA). Statistical significance was determined at *p* < 0.05.

## 5. Conclusions

According to the results, the effects of probiotics in combination are more potent than when taken alone, and this is probably because there is more butyric acid present. Therefore, we can assume that a combination supports the amplifier effect displayed by the combination by having a potentiated effect between the single probiotic component produced from distinct species. Moreover, the complex human internal environment remains difficult to create in an in vitro model, and future experiments are necessary to explore the link between microorganisms and human tissues/organs. The chosen probiotics point to their potential use in treating mood disorders in real-world settings after additional studies to explore the interaction with the gut–brain axis in a more complex system.

## Data Availability

The Laboratory of Physiology (C. Molinari) carefully stores raw data to ensure permanent retention under a secure system. This study’s data are available from the corresponding author upon reasonable request.

## Abbreviations

5HT	Serotonin
Adv DMEM	Dulbecco’s Modified Eagle’s Medium Advance
Adv DMEM-F12	Advanced Dulbecco’s Modified Eagle’s Medium/Nutrient F-12 Ham
AIWG	Antipsychotic-induced weight gain
BDNF	Brain-derived neurotrophic factor
CFU	Colony-forming unit
CNS	Central nervous system
Cyto-C	Cytochrome C
DMEM	Dulbecco’s Modified Eagle’s Medium
EMA	European Medicines Agency
FBS	Fetal bovine serum
FDA	Food and Drug Administration
GI	Gastrointestinal tract
GMB	Gut microbiota
HRP	Horseradish peroxidase
HTR1A	5-hydroxytryptamine receptor 1A
IBS	Irritable bowel syndrome
IL-1β	Interleukin-1β
IL-10	Interleukin-10
IL-6	Interleukin-6
L-Glu	L-Glutamic acid
M2	Muscarinic Acetylcholine Receptor
MTT	3-(4,5-Dimethylthiazol-2-yl)-2,5-diphenyltetrazolium bromide
PS128	*L. plantarum* PS128
SCFAs	Short-chain fatty acids
SCZ	Schizophrenia
TEER	Transepithelial electrical resistance
TMB	3,3′,5,5′-tetramethylbenzidine
TNFα	Tumor Necrosis Factor α
